# Platelet association with leukocytes in active eosinophilic esophagitis

**DOI:** 10.1371/journal.pone.0250521

**Published:** 2021-04-23

**Authors:** Kelly A. Bartig, Kristine E. Lee, Deane F. Mosher, Sameer K. Mathur, Mats W. Johansson

**Affiliations:** 1 Department of Biomolecular Chemistry, University of Wisconsin, Madison, Wisconsin, United States of America; 2 Department of Biostatistics and Medical Informatics, University of Wisconsin, Madison, Wisconsin, United States of America; 3 Department of Medicine, University of Wisconsin, Madison, Wisconsin, United States of America; 4 Morgridge Institute for Research, Madison, Wisconsin, United States of America; University of Michigan, UNITED STATES

## Abstract

We previously demonstrated that the percentage of blood eosinophils that are associated with platelets and thus positive for CD41 (integrin α_IIb_-subunit) correlates with and predicts peak eosinophil count (PEC) in biopsies of eosinophilic esophagitis (EoE) patients after treatment. Thus, flow cytometric determination of CD41+ eosinophils is a potential measure of EoE disease activity. Determinants of association of platelets with eosinophils and other leukocytes in EoE are largely unknown. The objectives of this study were to test the hypotheses that platelets associate with blood leukocytes other than eosinophils in EoE and that such associations also predict EoE activity. Whole blood flow cytometry was performed on samples from 25 subjects before and after two months of standard of care EoE treatment. CD41 positivity of cells within gates for eosinophils, neutrophils, monocytes, lymphocytes, and natural killer cells was compared. We found that percent CD41+ neutrophils, monocytes, and eosinophils correlated with one another such that principal component analysis of the five cell types identified “myeloid” and “lymphoid” factors. Percent CD41+ neutrophils or monocytes, or the myeloid factor, like CD41+ eosinophils, correlated with PEC after treatment, and CD41+ neutrophils or the myeloid factor predicted PEC < 6/high power field after treatment, albeit with lower area under the curve than for CD41+ eosinophils. We conclude that the processes driving platelets to associate with eosinophils in EoE also drive association of platelets with neutrophils and monocytes and that association of platelets with all three cell types is related to disease activity. Clinicaltrials.gov identifier: NCT02775045.

## Introduction

Eosinophilic esophagitis (EoE) is a chronic inflammatory disease that is characterized by symptoms of esophageal dysfunction and eosinophilic infiltration of the esophageal mucosa and has increased in incidence and prevalence during the last couple of decades [1,2]. The standard for assessing disease activity is endoscopy and pathological examination of esophageal biopsy specimens. There is an obvious need for biomarkers to replace such invasive and expensive monitoring. We recently demonstrated by flow cytometry that the percentage of blood eosinophils positive for CD41 (cluster of differentiation antigen 41, ITGA2B, α_IIb_ subunit of the major platelet-specific integrin) correlated with peak eosinophil count (PEC) per microscopic high power field (HPF) of esophageal biopsies from EoE patients after standard of care treatment [3]. We screened for 16 possible eosinophil-surface markers and CD41 was the one that correlated best with and predicted PEC [3]. Blood eosinophil CD41 positivity predicted after-treatment PEC < 6/HPF [3], a cutoff that has been used in various studies as a measure of remission of EoE [4,5]. We suggested, therefore, that platelet-eosinophil complexes as measured by percentage CD41+ eosinophils may be a useful biomarker of EoE activity [3]. Further, we observed immunohistochemical staining for platelets associated with eosinophils in vessels of EoE biopsies [3], supporting the presence *in vivo* of platelet-eosinophil complexes in EoE.

There is an extensive literature that platelets interact with neutrophils and monocytes as well as with eosinophils *in vivo* in allergic, inflammatory, cardiovascular and other diseases [6–18]. Platelet-leukocyte complexes have been observed and quantified in sections of lung biopsies from patients with asthma [19,20]. Studies, including intravital microscopy imaging, in a mouse inflammatory model indicate that neutrophils scan for, engage, and associate with activated platelets present in the bloodstream *in vivo* and that the P-selectin counter-receptor P-selectin glycoprotein ligand (PSGL)-1 on neutrophils transduces signals from this interaction driving neutrophil migration and initiating inflammation [21]. Platelet-neutrophil complexes are associated with skin pathology in psoriasis in humans and a mouse model [22]. Other examples of human patient studies include increased platelet-neutrophil complexes in sickle cell disease [23] and platelet-monocyte complexes associated with the development of acute respiratory distress syndrome [24]. Platelet-leukocyte complexes were increased in asthmatic patients during the late phase response after whole-lung antigen challenge [25]. Complexes between platelets and leukocytes, type unspecified, were associated with adverse events in patients undergoing surgery for rheumatic heart disease [26]. Results of the two latter studies raise the possibility that platelet associations with the different types of leukocyte increase together, even though association with individual types was not measured.

We reasoned that EoE, in which eosinophil infiltration is the hallmark of disease activity, is an informative condition to ask whether platelets associate with multiple leukocyte types even though one type dominates the inflammation. We therefore carried out additional analysis of the flow cytometry data that we used to assess platelet-eosinophil complexes in EoE [3] and quantified platelet association with blood neutrophils, monocytes, natural killer (NK) cells, and non-NK lymphocytes. We found that platelets associate with the four cell types, in addition to eosinophils, and that association with neutrophils and monocytes, as with platelet-eosinophil association, is related to EoE disease activity.

## Materials and methods

### Patients, study visits, and assessments

The protocol, approved by the University of Wisconsin (UW)-Madison Health Sciences Institutional Review Board, has been described previously [3]. Adult patients (> 18 years of age) with established EoE were recruited from the UW Health Gastroenterology Clinic following a diagnostic endoscopy for esophageal dysfunction. Patients with esophageal biopsy showing PEC of ≥ 15/HPF despite use of high dose proton pump inhibitor (PPI, equivalent of omeprazole ≥ 40 mg daily) for at least two months were invited to participate and enroll within two weeks of receiving the endoscopy results. At the enrollment visit (V1), informed consent was obtained along with a blood sample. Patients with concomitant allergic rhinitis completed the Rhinitis Control Assessment Test (RCAT) [3]. Patients with concomitant asthma had spirometry performed and completed the Asthma Control Questionnaire-7 [3]. Allergy skin prick testing for aeroallergens and foods was performed at V1. Patients received standard of care EoE treatment for eight weeks, swallowed topical steroid or food elimination as deemed most appropriate for a patient. At the follow-up visit (V2) at 8 weeks, repeat diagnostic endoscopy and blood sampling were performed. PEC was defined as the greatest value in biopsies from either the proximal or distal esophagus. At both visits, white blood cell counts were performed on heparinized whole blood using a Beckman Coulter Z1 particle counter.

Twenty-five patients completed the study between 2016 and 2018. These included two patients who were enrolled with protocol deviations. One had a contraindication to PPI use and did not undergo the lead-in trial with a PPI. The second had a PEC of 11/HPF at the time of enrollment. At V2, PEC < 6/HPF was used as the cutoff, a criterion for remission that has been used in various studies [4,5]. Eleven of the 25 patients (44%) had PEC < 6/HPF at V2. The mean age of the 25 patients was 38 years (standard deviation 14 years). Nineteen (76%) of the patients were male. Twenty-four (96%) of the patients were White non-Hispanic. One patient was White and American Indian/Alaska Native [3].

### Flow cytometry

**Fig 1:**
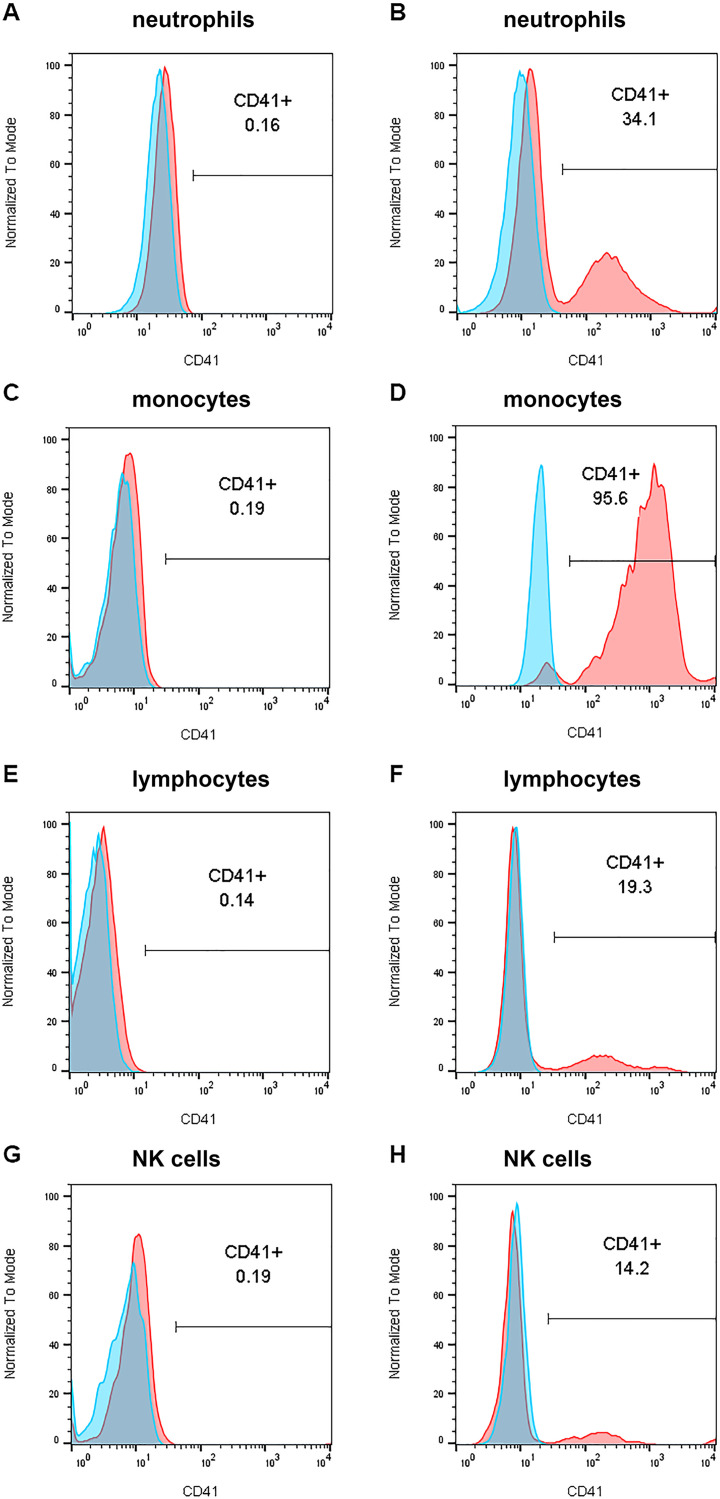
Examples of flow cytometry histogram for blood leukocyte CD41 at V2. Red, CD41; blue, isotype control. A and B) Neutrophils. C and D) Monocytes. E and F) Lymphocytes. G and H) Natural killer (NK) cells. A, C, E, and G) Subject No. 8 with 0.2% CD41-positive neutrophils, monocytes, and NK cells; 0.1% CD41-positive lymphocytes; and PEC = 0/HPF. B, D, F, and H) Subject No. 13 with 34% CD41-positive neutrophils, 96% CD41-positive monocytes, 19% CD41-positive lymphocytes, 14% CD41-positive NK cells, and PEC = 85/HPF. Note that CD41+ cells in B, D, F, and H form a distinct peak to the right well separated from the CD41- cells to the left that overlay cells stained with non-immune control immunoglobulin. The location of the marker was set in the “valley” between the negative and positive peaks or subpopulations, as described in Methods section.

Venous blood from V1 and V2 was drawn into vacuum tubes containing CTAD (citrate, theophylline, adenosine, and dipyridamole) anticoagulant solution (BD Vacutainer Systems, Franklin Lakes, NJ), in order to minimize platelet activation. Flow cytometry of the whole, unfractionated CTAD blood was performed with antibodies and data acquisition at the UW Carbone Cancer Center Flow Cytometry Facility as described [3,27–29]. Briefly, 100 μl blood was directly incubated with 0.5 μg unlabeled primary anti-CD41 monoclonal antibody (mAb), clone HIP8 (BD Biosciences, San Jose, CA) [3], or mouse immunoglobulin (Ig) G_1_ isotype control, clone MOPC-21 (BD) [3], for 30 minutes at room temperature. This was part of a screening with multiple primary antibodies, some of which were not available as directly labeled reagents [3]. Samples were washed by addition of 1 ml phosphate-buffered saline (PBS) and centrifugation for 10 minutes at 260 x *g* (1,200 revolutions per minute in a Sorvall Technospin R centrifuge, Du Pont, Wilmington, DE), resuspended in 250 μl phycoerythrin (PE)-conjugated goat anti-mouse IgG secondary antibody (BD) [3] diluted 1:100 in FACS buffer (PBS with 2% bovine serum albumin [BSA] and 0.2% NaN_3_). After incubation for 30 minutes, samples were washed again with PBS, resuspended in 100 μl FACS buffer with 5 μl fluorescein isothiocyanate (FITC)-conjugated anti-CD14 mAb, clone M5E2, and 5 μl FITC-anti-CD16, clone 3G8 (both from BD) [3,30], and incubated for 30 minutes. Red blood cells (RBC) were lysed by incubation with 2 ml FACS lysing solution (BD) for 10 minutes followed by centrifugation. Incubations were at room temperature until after RBC lysis and then at 4°C. Samples were washed with 500 μl FACS buffer, resuspended in FACS fixative (1% paraformaldehyde, 67.5 mM Na cacodylate, 113 mM NaCl, pH 7.2), stored at 4°C, washed with 1 ml PBS, and resuspended in FACS buffer just before data collection [3,29]. Mid-range one-peak rainbow fluorescent beads (Spherotech, Lake Forest, IL) were run at setup to set the sensitivity of the detectors in a standardized manner, thus maximizing reproducibility and optimizing data comparisons among subjects and visits. Compensation was performed with unlabeled, FITC-labeled, and PE-labeled Calibrite (BD) beads [3]. Due to a switch-over from the FACS Calibur (BD Biosciences, San Jose, CA) to the Attune (ThermoFisher, Waltham, MA) by the Flow Cytometry Facility during the course of the study, data were collected on two different cytometers and made equivalent, as explained before [3]. Data analysis was performed using FlowJo (Ashland, OR). Scatter and staining with the FITC-anti-CD14 and anti-CD16 mAbs were used to gate leukocyte populations [3,28–30]. With the Calibur data, cells were first gated by regions within a side scatter-height (SSC-H) versus forward scatter (FSC)-H plot, then by gating those populations in a SSC-H versus FITC-H plot [3,28–30]. With the Attune data, cells were similarly first gated by SSC-H versus FSC-H, then for singlets by FSC-H versus FSC-area (A) [30] and SSC-H versus SSC-A, and then, similarly as for the Calibur data, by SSC-H versus the BL1 (FITC-anti-CD14/CD16)-H channel [3] (online Supporting Information section’s Fig 1, S1 Fig). Eosinophils were defined as SSC-high and CD14/CD16-negative, taking into account the auto-fluorescence in the FITC/BL1 channel; neutrophils as SSC-medium-high and CD16-positive; monocytes SSC-medium-low and CD14-positive; non-NK lymphocytes as SSC-low and CD14/16-negative; and NK cells as SSC-low and CD16-positive [3,28–30] (S1 Fig). Distributions of CD41 staining of the other leukocytes, as with eosinophils [3], were biphasic with a negative subpopulation overlapping with the isotype control to the left clearly separated from the positive population to the right (Fig 1), and data were expressed as percentage positive cells [3,28]. In addition, for V2, CD41 expression level data on the subpopulation of CD41-positive cells are presented as specific geometric mean channel fluorescence (gMCF) [3], versus the CD41-negative subpopulation. Further, overall CD41 level expression of a cell type was calculated as fraction (i.e., percentage/100) CD41-positive cells multiplied by the specific gMCF of the CD41-positive subpopulation.

### Statistical analysis

Original study data were collected and managed using REDCap (Research Electronic Data Capture) tools [31], hosted by the UW-Madison Department of Medicine [3]. Group data are shown as median with 25^th^ and 75^th^ percentiles and/or range. Pearson test or Spearman rank test was used to analyze correlations, depending whether all variables were normally distributed or not, respectively. Paired *t* or Wilcoxon matched-pairs signed-rank test was used to compare data between two visits by the same subjects, depending on whether a variable was normally distributed or not. Mann-Whitney *U* test was used to compare data between two groups of subjects. χ2 test was used in contingency table analysis to compare nominal data between two groups. Principal component analysis (PCA) was performed at V1, V2, and with V2-V1 data in order to identify orthogonal (not correlated) factors that explain the variation in the data, using the procedure FACTOR, https://support.sas.com/documentation/onlinedoc/stat/141/factor.pdf in SAS (Cary, NC). Receiver operating characteristic (ROC) curve analysis was performed to evaluate the ability of CD41-positive cells or a PCA factor to predict PEC < 6/HPF at V2. Logistic regression was used to yield odds ratios representing the relative odds of having the outcome (PEC < 6/HPF) as percent CD41-positive cells increases (by 10% positive cells) or a PCA factor increases (by 1), reflecting the association of CD41-positive cells or a PCA factor to PEC. A level of probability (p) ≤ 0.05 was considered significant. Analyses were done and graphs generated using Prism (GraphPad, San Diego, CA), SAS, or Origin (Northampton, MA).

## Results

### Gating of leukocytes and quantification of CD41+ and CD41- populations for each cell type

Percent CD41+ neutrophils, monocytes, lymphocytes, or NK cells was determined by examining gatings (S1 Fig) in archived flow cytometry data gathered on whole blood samples from 25 UW-Madison EoE patients at visits 1 (V1) before and 2 (V2) eight weeks after standard of care EoE treatment [3]. Values were compared to the percent CD41+ eosinophil data from our previous study [3]. White blood cell counts at V1 and V2 and the distributions of cells among the five gatings are presented in S1 Table; median counts were within the normal range. There was no significant difference in cell populations between V1 and V2. Flow histogram examples of leukocyte CD41 positivity are shown in Fig 1. Note that CD41+ cells form a distinct peak to the right that is well separated from the CD41- cells to the left, the latter of which overlay cells stained with non-immune control immunoglobulin (Fig 1B, 1D, 1F and 1H). The location of the boundary between negative and positive cells was set to the nadir or “valley” between the two peaks, as described above under Methods and previously for eosinophils [3,28].

### CD41 positivity of leukocytes and correlations

**Fig 2:**
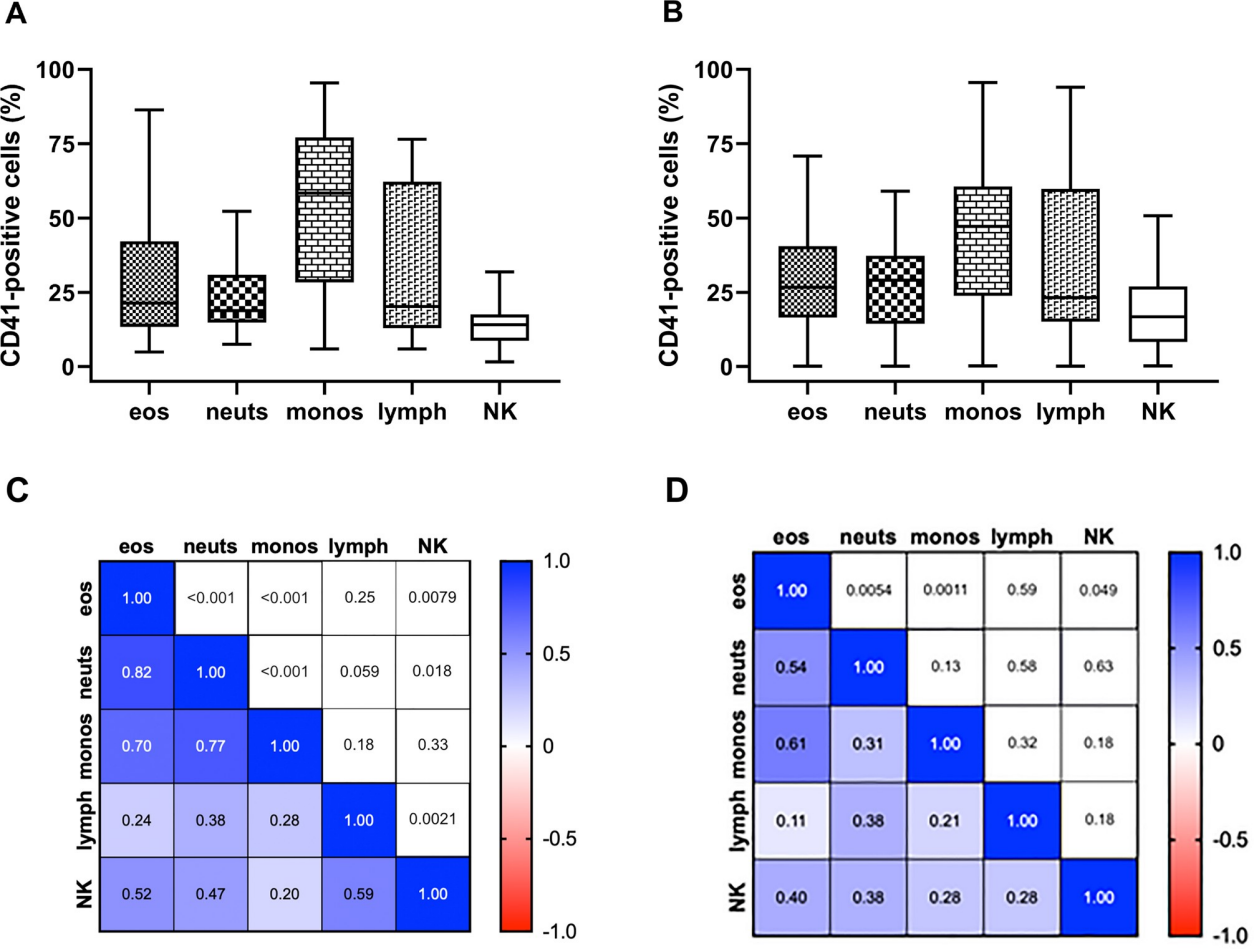
Blood leukocyte CD41 and correlations among leukocytes at V1 and V2. A and B) CD41-positive leukocytes at V1 (A) and V2 (B), median and quartiles. C and D) Correlations among the various leukocytes at V1 (C) and V2 (D). Numbers in colored lower left area represent Spearman rank (in C) and Pearson (in D) correlation coefficient values, respectively. Numbers in white upper right area represent p values. Eos, eosinophils; lymph, lymphocytes; monos, monocytes; neuts, neutrophils; NK, natural killer cells.

All five leukocyte populations contained cells that were positive for CD41 indicative of platelet association. There was considerable variability (i.e., high coefficient of variation, CV) among individual subjects for all cell types at V1 and V2 (Fig 2A and 2B and S2 Table). The CD41 expression level of the CD41+ subpopulations analyzed at V2 varied less among subjects than did percentage CD41+ cells, e.g., for eosinophils a CV of 14% for the CD41 level of the positive cells (S3 Table) compared to a CV of 62% for percent positivity (S2 Table). CD41 level of CD41-positive cells correlated with percentage CD41 positivity for lymphocytes but not for the other cell types (S3 Table). Calculated mean CD41 level for all cells of a particular leukocyte type correlated strongly with percent CD41 positivity for that cell type (S3 Table).

Changes in CD41 positivity in individual subjects from V1 to V2 are shown in S2 Fig. In 18 of the 25 subjects, percent CD41+ neutrophils and monocytes changed in the same direction as CD41+ eosinophils (in 24 subjects neutrophils and in 19 subjects monocytes changed in the same direction as eosinophils). Of these 18 subjects, the positivity of the three myelocytic types decreased in twelve and increased in six. The directions of changes in percent CD41+ NK cells and non-NK cell lymphocytes were the same as those for the three myelocytic types in 12 and 13 subjects, respectively (and were the same as eosinophils in 17 and 16 subjects, respectively).

Percent CD41+ neutrophils, monocytes, and eosinophils correlated among each other at V1 (Fig 2C), and neutrophils and monocytes correlated with eosinophils at V2 but not with one another (Fig 2D). Three-dimensional (3D) plots of percent positivities of the three myeloid cell types demonstrate the loosening of the correlations from V1 to V2 (S3 Fig). NK cells correlated with eosinophils at V1 and V2 (Fig 2C and 2D) and with lymphocytes and neutrophils at V1 (Fig 2C). Comparing V1 and V2 samples as groups, the percentages of CD41+ cells ranged lower at V2 to ≈ 0% (Fig 2B), but the V1 and V2 CD41+ cell values were not significantly different (S2 Table). In contrast, PEC decreased significantly during treatment, from V1 to V2 (S2 Table) [3]. The changes in CD41+ cells during the treatment period, i.e., V2 values minus V1 values, are displayed in S4 Fig.

### Principal component analysis (PCA) identifies a “myeloid” and a “lymphoid” factor

**Fig 3:**
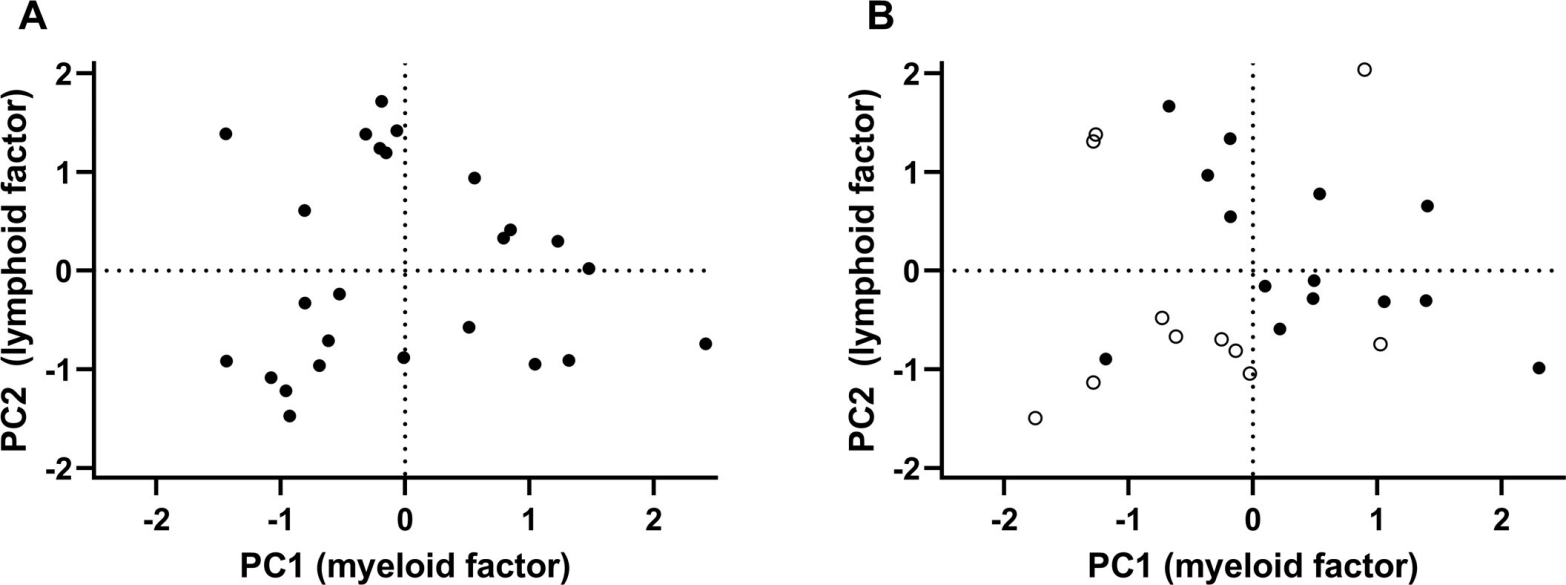
Principal component analysis (PCA) plots. Principal component (PC) 2 (lymphoid factor) (on y axis) versus PC2 (myeloid factor) (on x axis). A) At V1. B) At V2, empty symbols, PEC < 6/HPF.

Percent CD41+ values of the leukocyte populations at each visit and the changes from V1 to V2 were analyzed by PCA, leading to the identification of two factors. Factor 1, designated the “myeloid factor”, primarily used the information from eosinophils, neutrophils, and monocytes with roughly equal weights for these three cell types. Factor 2, designated the lymphoid factor, primarily used the information from lymphocytes and NK cells with negative weight from eosinophils and negligible impact from neutrophils and monocytes. Fig 3 shows PCA plots at V1 and V2.

### Percent CD41+ eosinophils, neutrophils, and monocytes, and the myeloid factor (PCA factor 1) correlate with PEC at V2

Of the 25 subjects, repeat biopsies demonstrated remission of EoE at V2 with PEC < 6 in 11 subjects (44%) and percent CD41+ eosinophils correlated with PEC at V2 [3]. Thus, we asked if CD41 positivity of the other four cell types and the PCA factors correlated with PEC at V2. We found significant correlations for neutrophils, monocytes, and the myeloid factor (Table 1, S5 Fig). Adjustment for treatment (swallowed topical steroid and/or food elimination) did not affect the correlations between eosinophil, neutrophil, or monocyte CD41 positivity, or the myeloid factor with PEC at V2 (after adjustment Spearman rank correlation coefficient [r_s_] = 0.61, p = 0.002 for eosinophils; r_s_ = 0.42, p = 0.04 for neutrophils; r_s_ = 0.50, p = 0.02 for monocytes; and r_s_ = 0.54, p = 0.007 for the myeloid factor). Adjustment for rhinitis (RCAT score) or allergy and asthma did not affect the correlations between eosinophil CD41 positivity or the myeloid factor with PEC at V2 (S4 Table). Three-dimensional plots of the eosinophils, neutrophils, and PEC at V1 and V2 are displayed in S6A and S6B Fig, respectively. Points for which PEC < 6/HPF at V2 cluster in one volume of the plot (S6B Fig, red). Similarly, most subjects with PEC < 6/HPF cluster in the left part (with the lowest PCA factor 1 or myeloid factor) of the PCA plot for V2 (Fig 3B). In consistency with this, median myeloid factor for the 11 patients with PEC < 6/HPF at V2 was -0.62 (quartiles -1.29 and -0.02) and was significantly lower than median myeloid factor for the 14 patients with PEC > 6/HPF, which was 0.36 (-0.23,1.14) (p = 0.02). PCA factor 2/lymphoid factor was not significantly different between the two groups (medians -0.70 [-1.04, 1.31] and -0.13 [-0.38, 0.83], respectively, p = 0.15). In S3 Fig, data are shown in plots divided into those from group A (“PEC-low” at V2), i.e., the 11 subjects (44%) with PEC < 6/HPF at V2 (S2A Fig), and those from group B (“PEC-high” at V2), i.e., the 14 (55%) subjects with PEC > 6 (S2B Fig). The data from the groups A and B are summarized in S5 Table. In group A (“PEC-low”), PEC was significantly lower at V2 than at V1. At V2, PEC was significantly lower in group A than in group B (the “PEC-high” group), as expected since group A was defined as subjects with PEC < 6/HPF at V2. Similarly, in group A percent CD41+ blood eosinophils was lower at V2 than at V1, and at V2 eosinophil CD41 positivity was lower in group A than in group B. CD41+ neutrophils at V2 were, like CD41+ eosinophils, lower in group A than B. Monocyte CD41 positivity in group A was, also like CD41+ eosinophils, lower at V2 than at V1. CD41 level of the CD41-positive leukocytes did not correlate with PEC at V2 (S3 Table). However, mean CD41 level of eosinophils or neutrophils correlated with PEC at V2 (S3 Table), not surprisingly since the overall CD41 level correlated very strongly with percentage CD41-positive cells (S3 Table), as described above.

**Table 1:** Correlations between leukocyte CD41 positivity or principal component analysis (PCA) factors and PEC at V2.

Cell type	r_s_	p
Eosinophils	0.60	0.002
Neutrophils	0.40	0.05
Monocytes	0.43	0.03
Lymphocytes	0.18	0.39
NK cells	0.23	0.26
Myeloid factor	0.53	0.006
Lymphoid factor	0.19	0.36

Abbreviations: CD, cluster of differentiation; NK, natural killer; p, probability; PEC, peak eosinophil count; r_s_, Spearman rank correlation coefficient; V, visit.

### Percent CD41+ neutrophils or the myeloid factor is associated with and predicts PEC at V2

**Fig 4:**
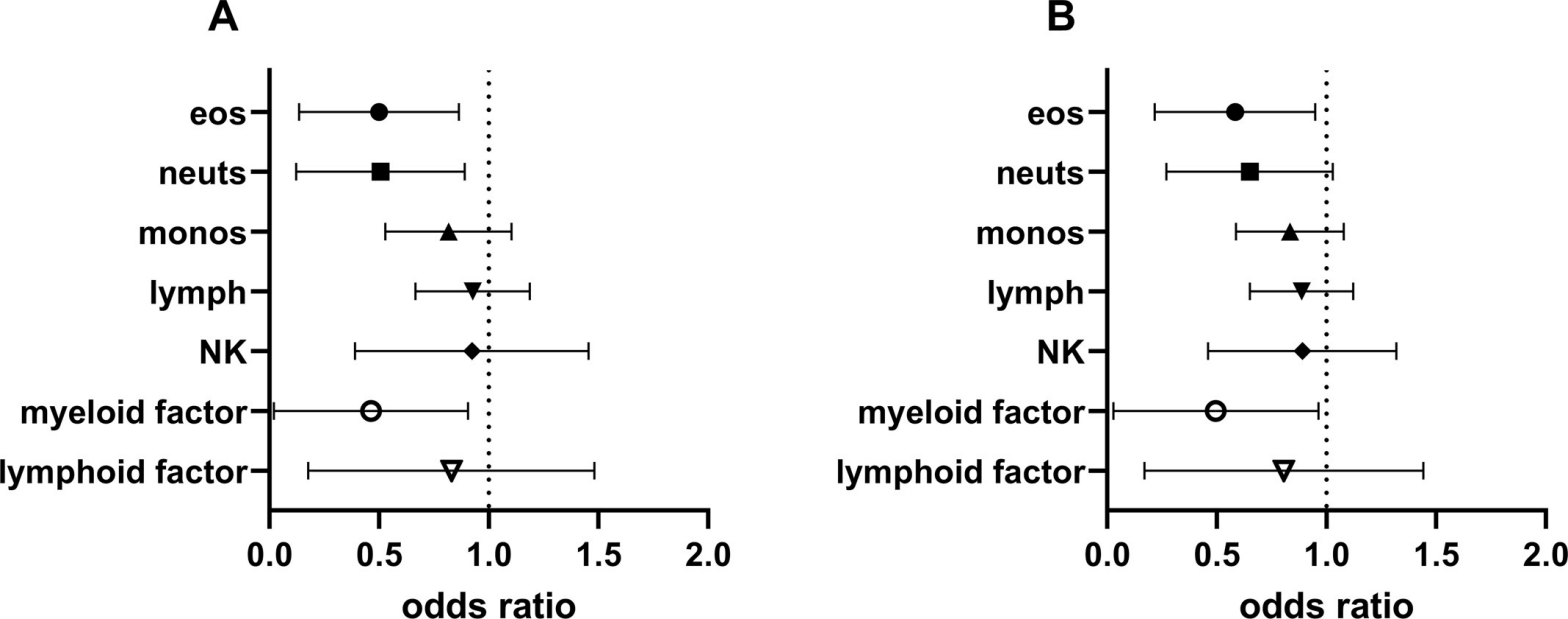
Odds ratios of CD41-positive leukocytes or PCA factors for PEC < 6 at V2. Odds ratios and 95% confidence intervals from logistic regression. A) With V2 data, p for eosinophils = 0.02, neutrophils = 0.03, monocytes = 0.18, lymphocytes = 0.48, NK cells = 0.52, myeloid factor = 0.04, lymphoid factor = 0.34. B) With V2-V1 data, p for eosinophils = 0.04, neutrophils = 0.08, monocytes = 0.16, lymphocytes = 0.28, NK cells = 0.43, myeloid factor = 0.06, lymphoid factor = 0.30. Eos, eosinophils; lymph, lymphocytes; monos, monocytes; neuts, neutrophils; NK, natural killer cells; PCA, principal component analysis.

Logistic regression produced significant odds ratios with confidence intervals < 1 for CD41-positive eosinophils and neutrophils as well as for the myeloid factor representing the relative odds of having PEC < 6/HPF at V2 as the percent CD41+ cells or the factor increases (Fig 4A). Thus, CD41-positive neutrophils, like eosinophils, and the myeloid factor were associated with PEC in such a way that an increasing value at V2 was associated with decreased odds for PEC < 6 at V2. In addition, the change in CD41-positive eosinophils from V1 to V2 had a significant odds ratio for PEC < 6/HPF at V2 (Fig 4B), indicating that the change in CD41+ eosinophils from V1 to V2 is significantly associated with the odds for PEC < 6 at V2. In addition, there were trends for the change in CD41-positive neutrophils and the myeloid factor to be associated with odds for PEC < 6 at V2 (Fig 4B).

**Fig 5:**
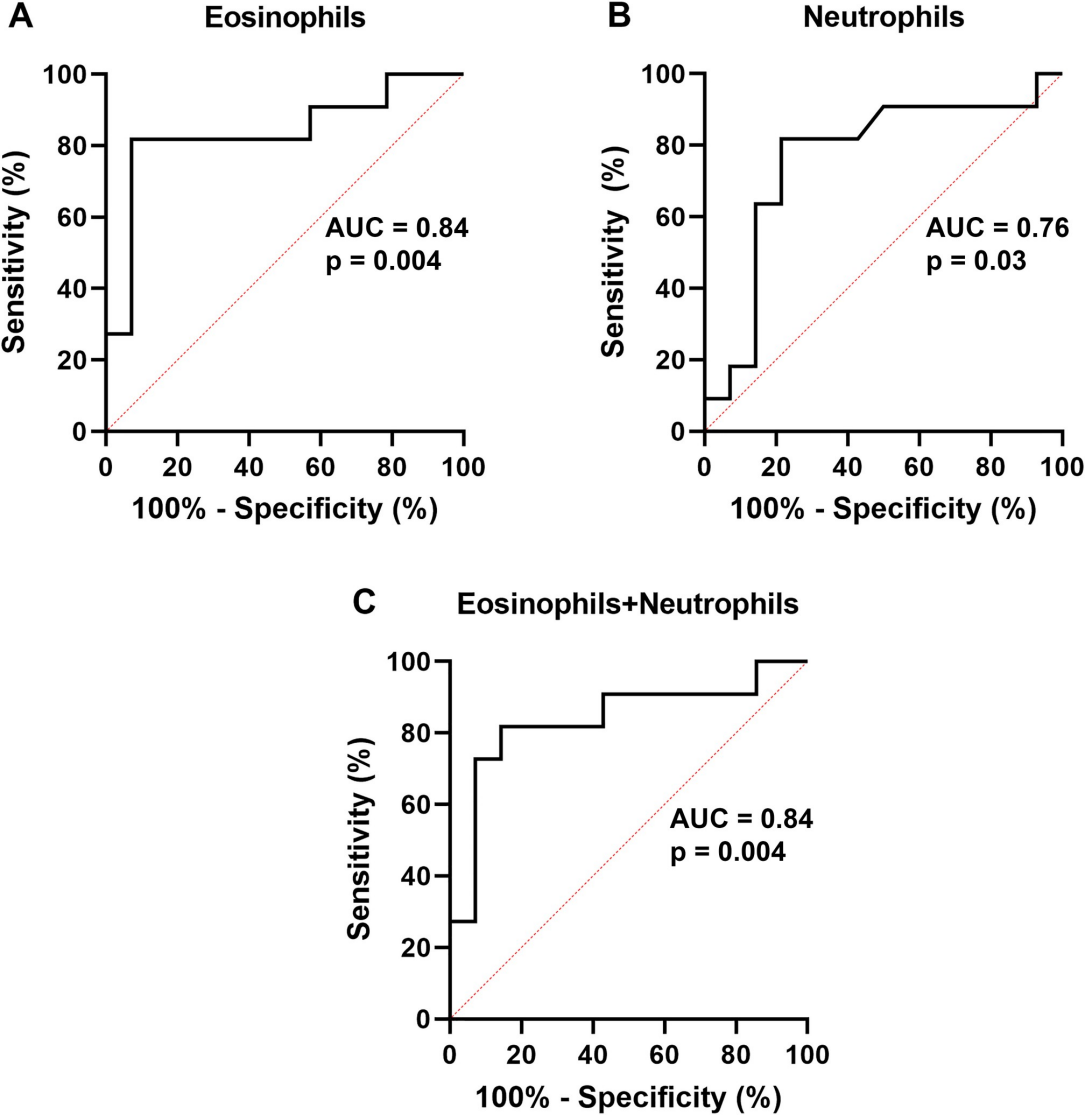
Receiver operating characteristic (ROC) curves for CD41-positive leukocytes to predict PEC < 6 at V2. A) Eosinophils, B) neutrophils, C) mean of eosinophil and neutrophil values. AUC, area under curve.

We performed ROC analysis to investigate the ability of CD41 positivity of other leukocytes, like eosinophil CD41 [3] (Fig 5A), to predict PEC < 6/HPF at V2, a cutoff that has been used in various studies as a measure of remission of EoE [4,5]. Neutrophil CD41 significantly predicted PEC with an area under curve (AUC) of 0.76 and a confidence interval that did not include 0.5, and with a statistically optimal criterion of < 27.8% CD41+ cells (Table 2 and Fig 5B). Monocyte CD41 did not reach significance as a predictor of PEC with a confidence interval that overlapped 0.5 (Table 2). Simplistically combining eosinophil and neutrophil CD41 positivity by using the mean of both signals yielded a combined metric that predicted PEC with an AUC of 0.84, identical to that of eosinophils alone, with a criterion of < 23.5% mean eosinophils and neutrophil CD41 positivity (Table 2 and Fig 5C). Similarly, the myeloid factor (PCA factor 1) significantly predicted PEC with an AUC of 0.77 and confidence interval that did not overlap 0.5 (Table 3).

**Table 2:** ROC analysis for the ability of percentage CD41-positive leukocytes to predict PEC < 6 at V2.

Variable	Eosinophils	Neutrophils	Monocytes	Lymphocytes	NK cells	Eosinophils + neutrophils
AUC	0.84	0.76	0.68	0.64	0.61	0.84
Confidence interval	0.66, 1.00	0.55, 0.97	0.45, 0.90	0.38, 0.89	0.37, 0.85	0.66, 1.00
P	0.004	0.03	0.14	0.25	0.35	0.004
Cutoff (% CD41 positive)	< 22.9	< 27.8	< 48.4	< 21.8	< 13.0	< 23.5

Abbreviations: AUC, area under curve; CD, cluster of differentiation; NK, natural killer; p, probability; PEC, peak eosinophil count; V, visit.

Note: Eosinophils + neutrophils, mean of percentage CD41-positive eosinophils and neutrophils.

**Table 3:** ROC analysis for the ability of principal component analysis (PCA) factors to predict PEC < 6 at V2.

Variable	Myeloid factor	Lymphoid factor
AUC	0.77	0.68
Confidence interval	0.57, 0.96	0.42, 0.93
P	0.02	0.14

Abbreviations: AUC, area under curve; PEC, peak eosinophil count; ROC, receiver operating characteristic; V, visit.

## Discussion

We found that platelets associate with blood leukocytes other than eosinophils in patients with EoE before and after standard of care treatment. Percent CD41+ neutrophils, monocytes, or NK cells correlated with percent CD41+ eosinophils. CD41 positivity of eosinophils, neutrophils, and monocytes correlated strongly among each other, and PCA identified a “myeloid” factor that used the information from eosinophil, neutrophil, and monocyte CD41 positivity.

Percent CD41+ neutrophils or monocytes, or the myeloid PCA factor, like percent CD41+ eosinophils, correlated with PEC at V2 after EoE treatment. These correlations were not affected by adjustment for treatment. Further, CD41 positivity of neutrophils and the myeloid factor significantly predicted, by ROC analysis, response in PEC to treatment. Combining percent CD41+ neutrophils and eosinophils in a simple manner did not give additional predictive value compared to eosinophils alone. Finally, logistic regression analysis demonstrated that an increasing value of eosinophil or neutrophil CD41 positivity, or the myeloid factor after treatment was associated with lower odds for PEC < 6/HPF after treatment. In addition, regression showed that the change in CD41+ eosinophils from before to after treatment was associated with PEC < 6 after treatment. Thus, a patient whose eosinophil CD41 positivity signal decreased during treatment was more likely to have a low PEC (< 6/HPF) after treatment and the more the CD41 signal decreased, the more likely the patient was to have PEC < 6 after treatment.

A major limitation of the present study is the operational nature of the determination of platelet-leukocyte complex formation, which included several incubations and washes before fixation and flow cytometry data collection. Thus, one must extrapolate our data with reservations to estimates of the proportion of leukocytes complexed to platelets subjected to different flow regimes *in vivo*. However, we believe based on published information described in the Introduction that platelet-leukocyte complexes exist *in vivo* and that *in vitro* determination of complexes reflects the *in vivo* events, even though the numbers may not be exactly the same. A second limitation is the relatively small number of samples, 25 before and after treatment of EoE. To validate and extend our findings, a larger study is needed. We expect that the correlations in percent CD41 positivity among leukocytes and the identification of a major myeloid factor by PCA found in our subjects will be readily apparent in a study of similar size as ours, inasmuch as strong correlations in positivity were found in both V1 and V2 samples. A larger study, however, likely will be needed to assess the disease associations. Although the response rate of 44% (11 of 25 subjects) in our study is comparable to the experience of others treating EoE [32], we do not know whether eight weeks is optimal for determination of disease responsiveness and how percent CD41+ eosinophils, neutrophils, and monocytes, and the myeloid PCA factor change during the treatment period. Such information would be helpful in planning how large a study is needed with sufficient power to confirm or refute the conclusion that percent CD41+ eosinophils or neutrophils, or the myeloid factor are predictors of and associated with disease response. The variability of the CD41 positivity of leukocytes might be taken as a limitation. PEC was also variable among patients, particularly at V2, with only a proportion of patients going into remission as determined by histology. Because the variabilities of CD41 signals and PEC enabled associations among them to be revealed, we regard the variabilities in the CD41 positivity data to be an important, albeit poorly understood, attribute. Yet another potential limitation is that anti-CD16 was used in gating of eosinophils to exclude neutrophils, which express a high level of CD16. Although this is a standard gating method and eosinophils in general do not express CD16 [33,34], CD16 may be induced on a subset of eosinophils *in vitro* by various mediators or detected on a subset of eosinophils in allergic rhinitis or asthma or even healthy subjects, according to some reports but not others [33–40]. We cannot rule out that a small proportion of eosinophils was gated out in our subjects. This potential limitation is important, because the concentration of eosinophil progenitor cells in the circulation identified by flow cytometry has been demonstrated to correlate with tissue pathology measured by the EoE histology scoring system and is higher in and predicts active EoE [41,42].

The finding of a principal component consisting of platelet-eosinophil, -neutrophil, and -monocyte complexes raises the question of whether platelet association with neutrophils and monocytes as well as with eosinophils has clinical significance in EoE. We envision platelet activation as an upstream event leading to platelet-leukocyte complex formation, leukocyte activation, and “licensing” of leukocyte recruitment into tissues. Further, we hypothesize that platelet activation *in vivo* occurs to variable degrees among patients, the mechanism of or explanation for which remains to be investigated, resulting in variable degrees of platelet-leukocyte complexes. Because platelets are generally 30- to 50-fold greater in number than leukocytes in blood, only a minority of platelets would need to be activated to initiate such a pathway. We observed immunohistochemical staining for platelets associated with eosinophils in vessels of EoE biopsies [3] and we earlier demonstrated in *in vitro* experiments that interaction of P-selectin, which is expressed on the surface of activated platelets, with eosinophils activates eosinophil α_4_β_1_ integrin [28]. Selective eosinophil recruitment in EoE compared to other leukocytes, therefore, may be favored by α_4_β_1_ integrin-mediated arrest on vascular cell adhesion molecule-1 (VCAM1), which is upregulated on activated endothelium in EoE [43]. Further, eosinophils may be primed to migrate toward chemoattractants with specificity for eosinophils, such as eotaxin-3 (CCL26) [43–45]. Whether associations between neutrophils or monocytes and platelets also can be observed in EoE biopsies remains to be investigated, along with analyses of molecules and adhesion receptors to call these leukocytes into esophageal tissues. Regarding the concept of platelet activation in EoE, a recent interesting finding may be relevant: Plasminogen activator inhibitor (PAI)-1, a risk factor for thrombosis and the principal inhibitor of urokinase, in turn an activator of plasminogen and hence of fibrinolysis, was reported to be elevated in the epithelium of patients with active EoE [46].

That platelets associate with neutrophils in EoE in a manner that is associated with EoE disease activity is consistent with reports on platelet-neutrophil complexes in other inflammatory as well as infectious diseases [7,8,10,15,18,21–23,47]. Although percent CD41+ neutrophils or the myeloid factor predicted PEC and was associated with PEC by regression analysis, with somewhat lower predictive value than eosinophils, neutrophils are generally ten-fold more abundant in blood than eosinophils. Thus, it may be possible to devise a non-flow cytometric assay for EoE disease activity based on the more numerous platelet-neutrophil complexes that does not require the incubations and washes of a flow cytometry protocol. In particular, a microfluidic drop of blood assay using neutrophil interaction with P-selectin has been developed as a potential biomarker for asthma [48].

The demonstrations that platelets associate with various blood leukocyte types in EoE and of a principal myelocyte component or factor informed by platelet association with eosinophils, neutrophils, and monocytes raise the question of whether the factor can be detected in diseases that are associated with platelet-neutrophil and platelet-monocyte complexes. Such findings would implicate the platelet as a driver of leukocyte behavior in multiple conditions and perhaps provide an explanation for the association of disease processes such as atherosclerosis and asthma [49–52].

## Supporting information

S1 Fig Gating of blood eosinophils, neutrophils, monocytes, lymphocytes, and NK cells. Populations in whole blood leukocytes were first gated based on SSC-H vs FSC-H (top dot plot) with gate A (high SSC, intermediate FSC) encompassing eosinophils, gate B (medium-high SSC, intermediate FSC) encompassing neutrophils, gate C (medium-low SSC, intermediate FSC) encompassing monocytes, and gate D (low SSC, low FSC) including lymphocytes and NK cells. Rows 1–4, left and middle plots: Among cells from A-D, doublets were excluded based on FSC-H versus FSC-A and SSC-H versus SSC-A, respectively. Right plots: Singlets were gated further based on SSC-H versus FITC-anti-CD14/CD16 to include eosinophils (row 1, from A, CD14/CD16-negative, autofluorescent in the FITC/BL1 channel), neutrophils (row 2, from B, CD16-positive), monocytes (row 3, from C, CD14-positive), lymphocytes (row 4, from D, CD14/CD16-negative), and NK cells (row 4, from D, CD16-positive). A, area; FSC, forward scatter; H, height; lymph, lymphocytes; NK, natural killer cells; SSC, side scatter.
(TIF)

S2 Fig CD41-positive blood leukocytes at V1 and V2 in each subject. A) Group A or “PEC-low”, subjects with PEC < 6/HPF at V2. B) Group B or “PEC-high”, subjects with PEC > 6/HPF at V2. Eos, eosinophils (red); lymph, lymphocytes (brown); monos, monocytes (blue); neuts, neutrophils (green); NK, natural killer cells (black). Twenty-eight patients were initially enrolled. Subjects No. 12, 21, and 25 did not complete the study [3].
(TIF)

S3 Fig Three-dimensional plots visualizing the correlations among percentage CD41-positive eosinophils, neutrophils, and monocytes. (A) At V1. (B) At V2.
(TIF)

S4 Fig Changes in CD41-positive blood leukocytes from V1 to V2. Median, quartiles, and range are shown. Eos, eosinophils; lymph, lymphocytes; monos, monocytes; neuts, neutrophils; NK, natural killer cells.
(TIF)

S5 Fig Correlations between leukocyte CD41 positivity and PEC at V2. A) Eosinophils, B) neutrophils, C) monocytes, D) lymphocytes, E) NK cells.
(TIF)

S6 Fig Three-dimensional plots visualizing the correlations among percentage CD41-positive eosinophils and neutrophils, and PEC. A) At V1. B) At V2, red, PEC < 6.
(TIF)

S1 Table Blood cell counts at V1 and V2. (DOCX)

S2 Table Percentage CD41-positive leukocytes and PEC (median, quartiles, and CV) at V1 and V2. (DOCX)

S3 Table CD41 expression level on CD41-positive and all leukocytes, and correlations with percentage CD41 positivity and PEC, at V2. (DOCX)

S4 Table Correlations between leukocyte CD41 positivity or principal component analysis (PCA) factors and PEC at V2, adjusted for RCAT or allergy and asthma. (DOCX)

S5 Table Percentage CD41-positive leukocytes (median and quartiles) at V1 and V2 in subjects with PEC < or > 6/HPF at V2. (DOCX)
