# Supplementary figures and images for: Platelet association with leukocytes in active eosinophilic esophagitis
Source: PLoS One. 2021 Apr 23;16(4):e0250521. doi: 10.1371/journal.pone.0250521 (PMC8064567; doi:10.1371/journal.pone.0250521)

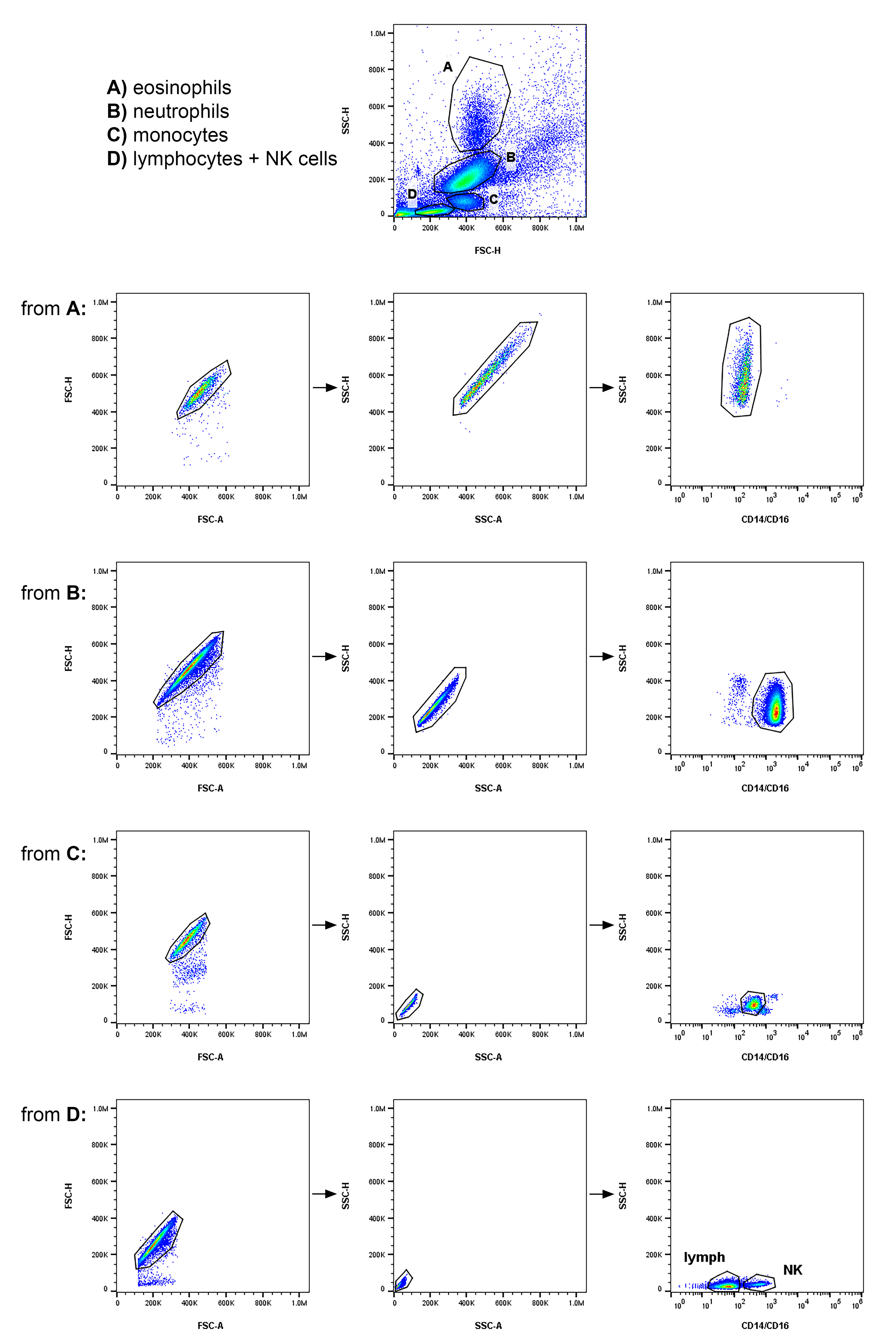

Supplement: S1 Fig — Populations in whole blood leukocytes were first gated based on SSC-H vs FSC-H (top dot plot) with gate A (high SSC, intermediate FSC) encompassing eosinophils, gate B (medium-high SSC, intermediate FSC) encompassing neutrophils, gate C (medium-low SSC, intermediate FSC) encompassing monocytes, and gate D (low SSC, low FSC) including lymphocytes and NK cells. Rows 1–4, left and middle plots: Among cells from A-D, doublets were excluded based on FSC-H versus FSC-A and SSC-H versus SSC-A, respectively. Right plots: Singlets were gated further based on SSC-H versus FITC-anti-CD14/CD16 to include eosinophils (row 1, from A, CD14/CD16-negative, autofluorescent in the FITC/BL1 channel), neutrophils (row 2, from B, CD16-positive), monocytes (row 3, from C, CD14-positive), lymphocytes (row 4, from D, CD14/CD16-negative), and NK cells (row 4, from D, CD16-positive). A, area; FSC, forward scatter; H, height; lymph, lymphocytes; NK, natural killer cells; SSC, side scatter. (TIF) [file pone.0250521.s001.tif]

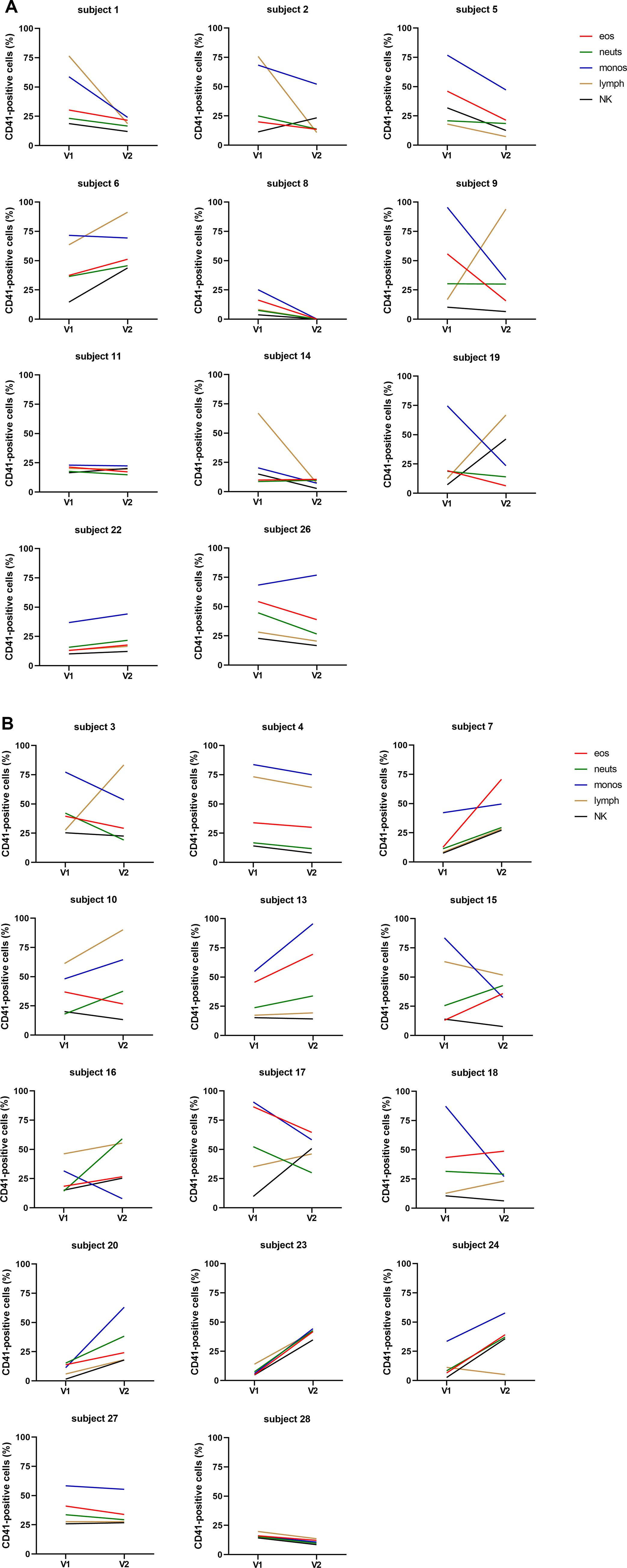

Supplement: S2 Fig — A) Group A or “PEC-low”, subjects with PEC < 6/HPF at V2. B) Group B or “PEC-high”, subjects with PEC > 6/HPF at V2. Eos, eosinophils (red); lymph, lymphocytes (brown); monos, monocytes (blue); neuts, neutrophils (green); NK, natural killer cells (black). Twenty-eight patients were initially enrolled. Subjects No. 12, 21, and 25 did not complete the study [3]. (TIF) [file pone.0250521.s002.tif]

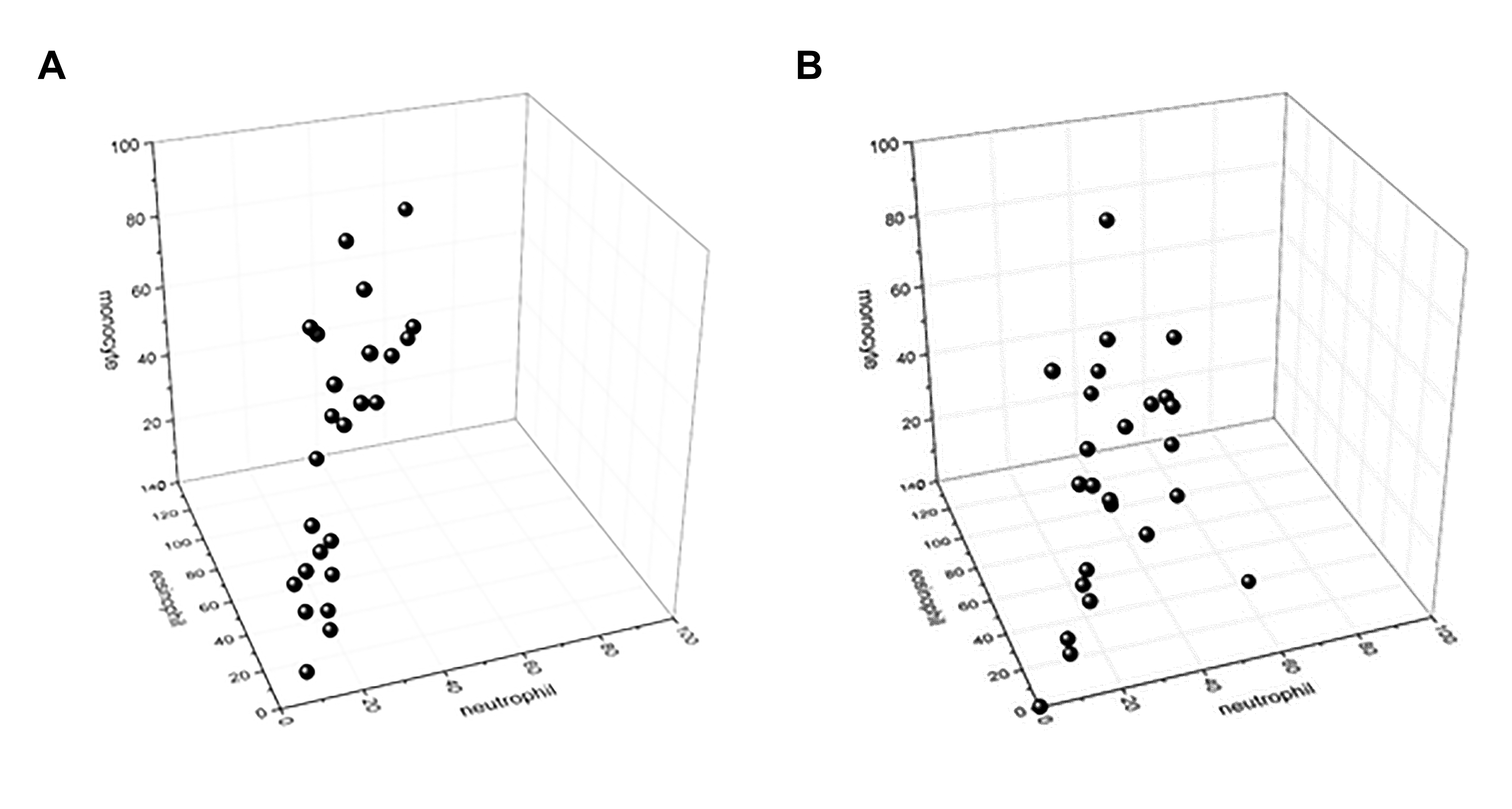

Supplement: S3 Fig — (A) At V1. (B) At V2. (TIF) [file pone.0250521.s003.tif]

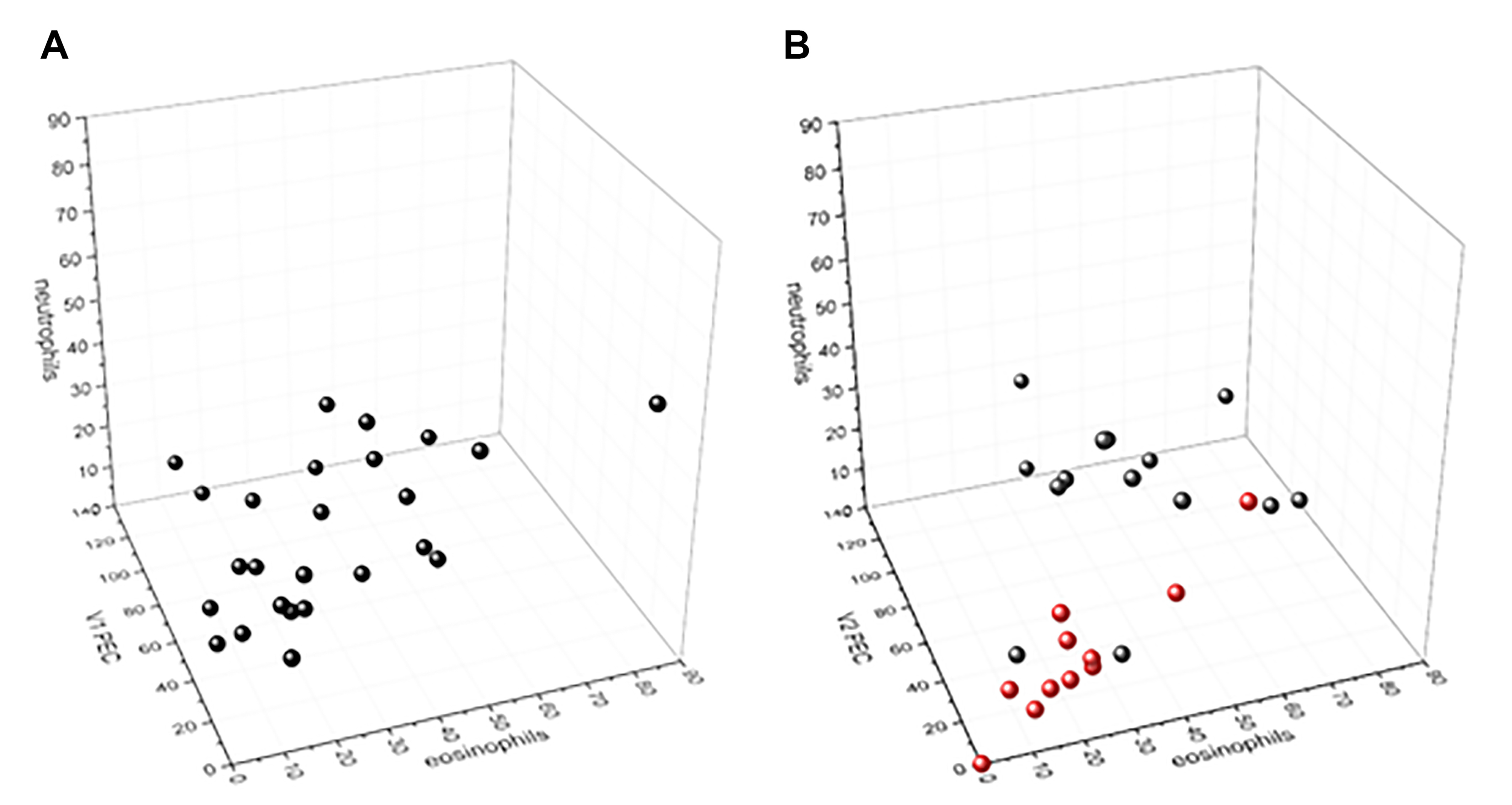

Supplement: S6 Fig — A) At V1. B) At V2, red, PEC < 6. (TIF) [file pone.0250521.s006.tif]
