# Supplementary material for: Platelet association with leukocytes in active eosinophilic esophagitis
Source: PLoS One. 2021 Apr 23;16(4):e0250521. doi: 10.1371/journal.pone.0250521 (PMC8064567; doi:10.1371/journal.pone.0250521)
Supplement: S1 Table — (DOCX) [file pone.0250521.s007.docx]

| **S1 Table. Blood cell counts at V1 and V2.** | | |
| --- | --- | --- |
| **Variable** | **V1**  **Median (range)** | **V2**  **Median (range)** |
| White blood cells (per ml) | 5.1 (3.4 – 8.8) | 5.3 (2.3 - 11) |
| Eosinophils (%) | 3.5 (0.7 - 12) | 3.3 (0.8 - 12) |
| Neutrophils (%) | 62 (45 – 87) | 66 (51 – 88) |
| Monocytes (%) | 8.9 (2.6 - 20) | 7.6 (3.2 - 23) |
| Lymphocytes (%) | 22 (1.4 - 40) | 21 (1.8 - 37) |
| NK cells (%) | 3.1 (0.8 – 8.7) | 2.8 (1.1 – 9.2) |
| Abbreviations: NK, natural killer: V, visit.  Note: Percentages were adjusted to a total of 100% for each sample (i.e., each subject at each visit). | | |
