# Supplementary material for: Platelet association with leukocytes in active eosinophilic esophagitis
Source: PLoS One. 2021 Apr 23;16(4):e0250521. doi: 10.1371/journal.pone.0250521 (PMC8064567; doi:10.1371/journal.pone.0250521)
Supplement: S2 Table — (DOCX) [file pone.0250521.s008.docx]

| **S2 Table. Percentage CD41-positive leukocytes and PEC at V1 and V2.** | | | |
| --- | --- | --- | --- |
| **Cell type or PEC** | **V1**  **Median (quartiles; CV)** | **V2**  **Median (quartiles: CV)** | **p** |
| Eosinophils | 21.3 (13.5, 42.2; 66%) | 26.7 (16.5, 40.5; 62%) | 0.90 |
| Neutrophils | 18.8 (14.8, 30.9; 53%) | 29.1 (14.4, 37.3; 52%) | 0.55 |
| Monocytes | 58.4 (28.4, 77.1; 51%) | 47.2 (23.8, 60.6; 56%) | 0.13 |
| Lymphocytes | 20.2 (13.0, 62.2; 74%) | 23.2 (15.0, 59.8; 81%) | 0.44 |
| NK cells | 14.1 (8.7, 17.6; 55%) | 16.7 (8.3, 27.0; 70%) | 0.41 |
| PEC (per HPF) | 42 (28, 68; 60%) | 12 (0, 44; 128%) | 0.02 |
| Abbreviations: CD, cluster of differentiation; CV, coefficient of variation, HPF, high power field; NK, natural killer; p, probability; PEC, peak eosinophil count; V, visit. | | | |
