# Supplementary material for: Platelet association with leukocytes in active eosinophilic esophagitis
Source: PLoS One. 2021 Apr 23;16(4):e0250521. doi: 10.1371/journal.pone.0250521 (PMC8064567; doi:10.1371/journal.pone.0250521)
Supplement: S3 Table — (DOCX) [file pone.0250521.s009.docx]

| **S3 Table. CD41 expression level on CD41-positive and all leukocytes, and correlations with percentage CD41 positivity and PEC, at V2.** | | | | | | |
| --- | --- | --- | --- | --- | --- | --- |
| **Cell type** | **CD41 level of CD41-positive cells**  **(specific gMCF vs the CD41-negative cells, median [quartiles; CV])** | **Overall CD41 level (fraction positive cells x level of CD41+ cells)**  **(specific gMCF, median [quartiles; CV])** | **Correlation between CD41 level of CD41+ cells and percentage CD41 positivity (r_s_/p)** | **Correlation between overall CD41 level and percentage CD41 positivity**  **(r_s_/p)** | **Correlation between CD41 level of CD41+ cells and PEC**  **(r_s_/p)** | **Correlation between overall CD41 level and PEC**  **(r_s_/p)** |
| Eosinophils | 1070 (970, 1150; 14%) | 290 (180, 450; 61%) | -0.34/0.10 | 0.98/<0.001 | -0.08/0.70 | 0.59/0.002 |
| Neutrophils | 1220 (1070, 1400; 19%) | 260 (200, 460; 61%) | 0.08/0.72 | 0.97/<0.001 | -0.10/0.65 | 0.45/0.02 |
| Monocytes | 1500 (1230, 1700; 28%) | 600 (280, 900; 64%) | -0.04/0.87 | 0.90/<0.001 | -0.08/0.69 | 0.36/0.08 |
| Lymphocytes | 1980 (1580, 2920: 33%) | 460 (210, 1690; 102%) | 0.62/0.001 | 0.97/<0.001 | -0.10/0.63 | 0.11/0.60 |
| NK cells | 2130 (1290, 2680; 34%) | 280 (170, 570; 85%) | -0.16/0.45 | 0.84/<0.001 | -0.45/0.02 | 0.04/0.85 |
| Abbreviations: gMCF, geometric mean channel fluorescence; CV, coefficient of variation; p, probability; PEC, peak eosinophil count; r_s_, Spearman rank correlation coefficient; V, visit; vs, versus. | | | | | | |
