# Supplementary material for: Platelet association with leukocytes in active eosinophilic esophagitis
Source: PLoS One. 2021 Apr 23;16(4):e0250521. doi: 10.1371/journal.pone.0250521 (PMC8064567; doi:10.1371/journal.pone.0250521)
Supplement: S4 Table — (DOCX) [file pone.0250521.s010.docx]

| **S4 Table. Correlations between leukocyte CD41 positivity or principal component analysis (PCA) factors and PEC at V2, adjusted for RCAT or allergy and asthma.** | | | | |
| --- | --- | --- | --- | --- |
| **Correlation adjusted for:** | **RCAT** | | **Allergy and asthma** | |
| **Cell type** | **r_s_** | **p** | **r_s_** | **p** |
| Eosinophils | 0.56 | 0.009 | 0.55 | 0.006 |
| Neutrophils | 0.32 | 0.16 | 0.34 | 0.11 |
| Monocytes | 0.38 | 0.09 | 0.40 | 0.06 |
| Lymphocytes | 0.07 | 0.76 | 0.12 | 0.58 |
| NK cells | 0.19 | 0.42 | 0.18 | 0.42 |
| Myeloid factor | 0.48 | 0.03 | 0.50 | 0.02 |
| Lymphoid factor | 0.08 | 0.71 | 0.12 | 0.58 |
| Abbreviations: CD, cluster of differentiation; NK, natural killer; p, probability; PEC, peak eosinophil count; RCAT, Rhinitis Control Assessment Test; r_s_, Spearman rank correlation coefficient; V, visit. | | | | |
