# Supplementary material for: Platelet association with leukocytes in active eosinophilic esophagitis
Source: PLoS One. 2021 Apr 23;16(4):e0250521. doi: 10.1371/journal.pone.0250521 (PMC8064567; doi:10.1371/journal.pone.0250521)
Supplement: S5 Table — (DOCX) [file pone.0250521.s011.docx]

| **S5 Table. Percentage CD41-positive leukocytes at V1 and V2 in subjects with PEC < or > 6/HPF at V2.** | | | | | | | | |
| --- | --- | --- | --- | --- | --- | --- | --- | --- |
|  | **Group A or “PEC-low”: Subjects with PEC < 6/HPF at V2 (n = 11)** | | **Group B or “PEC-high”: Subjects with PEC < 6/HPF at V2 (n = 14)** | | **p** | | | |
| **Cell type or PEC** | **V1**  **Median (quartiles)** | **V2**  **Median (quartiles)** | **V1**  **Median (quartiles)** | **V2**  **Median (quartiles)** | **Group A V2 vs V1** | **Group B V2 vs V1** | **V1 group B vs group A** | **V2 group B vs group A** |
| Eosinophils | 21.3  (16.3, 46.1) | 17.3  (10.5, 21.6) | 26.2  (12.9, 41.6) | 34.9  (26.7, 52.7) | 0.05 | 0.16 | 0.61 | 0.003 |
| Neutrophils | 20.9  (15.8, 30.3) | 16.7  (13.8, 26.6) | 17.4  (13.6, 32.0) | 32.0  (26.6, 39.4) | 0.17 | 0.16 | 0.73 | 0.03 |
| Monocytes | 68.4  (25.2, 74.6) | 33.8  (22.4, 52.1) | 51.5  (27.8, 83.6) | 54.4  (31.2, 63.4) | 0.02 | 0.90 | 0.81 | 0.15 |
| Lymphocytes | 20.2  (13.2, 67.0) | 18.8  (7.4, 66.8) | 23.8  (12.4, 50.0) | 35.0  (19.1, 57.6) | 0.58 | 0.06 | 0.54 | 0.27 |
| NK cells | 14.6  (10.1, 18.8) | 12.6  (6.6, 23.4) | 14.0  (6.9, 16.5) | 20.2  (8.4, 29.1) | 0.97 | 0.30 | 0.58 | 0.37 |
| PEC (per HPF) | 58 (28, 87) | 0 (0, 3) | 41 (23, 60) | 36 (23, 72) | 0.001 | 0.92 | 0.38 | < 0.001 |
| Abbreviations: CD, cluster of differentiation; HPF, high power field; n, number of subjects; NK, natural killer; p, probability; PEC, peak eosinophil count; V, visit; vs, versus. | | | | | | | | |
